# Electrokinetic Phenomena in Pencil Lead-Based Microfluidics

**DOI:** 10.3390/mi7120235

**Published:** 2016-12-15

**Authors:** Yashar Bashirzadeh, Venkat Maruthamuthu, Shizhi Qian

**Affiliations:** Department of Mechanical & Aerospace Engineering, Old Dominion University, Norfolk, VA 23529, USA; ybash001@odu.edu (Y.B.); vmarutha@odu.edu (V.M.)

**Keywords:** alternating current electroosmosis (ACEO), induced-charge electroosmosis (ICEO), dielectrophoresis (DEP), 3D electrode

## Abstract

Fabrication of microchannels and associated electrodes to generate electrokinetic phenomena often involves costly materials and considerable effort. In this study, we used graphite pencil-leads as low cost, disposable 3D electrodes to investigate various electrokinetic phenomena in straight cylindrical microchannels, which were themselves fabricated by using a graphite rod as the microchannel mold. Individual pencil-leads were employed as the micro-electrodes arranged along the side walls of the microchannel. Efficient electrokinetic phenomena provided by the 3D electrodes, including alternating current electroosmosis (ACEO), induced-charge electroosmosis (ICEO), and dielectrophoresis (DEP), were demonstrated by the introduced pencil-lead based microfluidic devices. The electrokinetic phenomena were characterized by micro-particle image velocimetry (micro-PIV) measurements and microscopy imaging. Highly efficient electrokinetic phenomena using 3D pencil-lead electrodes showed the affordability and ease of this technique to fabricate microfluidic devices embedded with electrodes for electrokinetic fluid and particle manipulations.

## 1. Introduction

Microfluidic technology has become an important multi-disciplinary field for controlling and sensing in areas as diverse as electronics and tissue engineering. Small volumes of solvent, samples and reagents have been transported through microchannels embedded in a chip, and, additionally, micro-electrodes of different materials have been used for manipulation, control and/or detection of fluids and samples [1,2]. 

Electrokinetic transport processes such as electroosmosis, induced charge electroosmosis (ICEO) and dielectrophoresis (DEP) using electric fields have become important techniques to manipulate fluids and particles in various microfluidic applications [3,4]. ICEO flow is a nonlinear electro-osmotic flow and typically occurs around a conductive surface or floating electrode under an external direct current (DC) or low frequency alternating current (AC) electric field [5]. Alternating current electroosmosis (ACEO) flow is generated at electrode surfaces subject to an applied AC electric field [6]. DEP refers to the motion of particles caused by dielectric polarization effects induced by a spatially non-uniform electric field [7]. The aforementioned electrokinetic phenomena, including ACEO, ICEO and DEP, have been widely used for pumping, mixing, trapping, focusing and separating samples/particles in various microfluidic applications [8,9,10]. To achieve the aforementioned electrokinetic phenomena, micro-electrodes must be fabricated within the microfluidic device for either applying the electric field (in ACEO and DEP applications) or inducing charges at the surface of the floating electrode (in ICEO applications). 

Several methods for fabricating micro-electrodes in microfluidic devices have been developed in the literature. Common methods use photolithography followed by metal deposition and the lift-off process [11,12]. A conventional deposition process is sputtering, and it requires expensive equipment to deposit the metal or alloy film on the substrate. Another common conventional process electroplates a superior metal on a base metal. Even though this process can be low in cost for a thin film of deposited metal, it is costly and time-consuming for thicker coats of metal. Uniformity of such coats on the base metal has also been an issue [13]. An alternative approach injects low melting point liquid metals or alloys in a mold aligned to the microchannel [14]. Doped silicon [15] and insulating posts have also been used to produce non-uniform fields generated by metal electrodes for DEP applications. Insulating posts require application of high voltages to the system [16], and the cost of fabrication using doped silicon is a concern [17]. 

Use of low-cost and biocompatible 3D electrodes possessing good mechanical properties has always been of great interest in microfluidics to simplify the laborious fabrication process and lower its cost while providing efficient outputs. Use of 3D carbon-based electrodes [18] can be suitable for this purpose. Glass-like 3D carbon electrodes, for example, have been used to manipulate particles and biomolecules under low voltages [19,20]. Glass-like carbon electrodes can be fabricated by the carbonization process [20]. To achieve this, a two-step photolithography process was used to fabricate the precursor SU-8 structures, which were then converted into 3D carbon electrodes through pyrolysis, heating the structures up to high temperatures (900 °C for SU-8) [21]. Low voltage DEP using carbon electrodes fabricated using this low-cost method combines advantages of metal electrodes and insulator-based DEP. Even though carbon electrodes are electrically more resistive than highly conductive metals such as gold, there are some advantages associated with them over metals. Carbon possesses excellent mechanical properties and polarizes at lower voltages while minimizing the possibility of sample electrolysis [17]. Therefore, accessible and ready-made yet low-cost allotropes of carbon can further facilitate microfluidic device fabrication. Sub-mm pencil-lead, mechanical pencil core made of graphite mixed with clay, sounds promising for achieving this goal. Electrically, graphite is a semi-metal, conductive in its basal plane and an insulator normal to the basal plane [22]. Its special properties have led to several applications in electronics, semiconductor and energy industries. Its high heat and electrical conductivity, durability, and stability have provided researchers with electrodes capable of working at a wide range of operating conditions. In small scale applications, graphite pencil cores are capable of playing important roles in paper-based electronics [23,24] and microfluidics [25,26]. Graphite rods are fabricated in various types and diameters ranging from 200 micrometers to a few millimeters suitable for microfluidic applications. They have been embossed in a channel substrate of a planar fuel cell or mounted in the machined cavity of a 3D array fuel cell to serve as electrodes [27].

The present paper uses 3D graphite pencil-leads to provide a straightforward single-step device fabrication method complementing other challenging conventional fabrication techniques that require multiple steps and processes. For instance, conventional photolithography techniques pattern expensive photoresists, such as SU-8, on clean surfaces as molds. These molds provide only vertical sidewalls when they are exposed to ultraviolet (UV) light and developed. As mentioned, conventional electrode fabrication processes deposit a thin layer of metal such as gold on a substrate surface to be used often as a planar electrode. Here, pencil-leads provided us with simple 3D side-wall electrodes that could be aligned with the microchannel. The major advantage of using 3D electrodes as bulky polarizable objects over planar conductive surfaces is the generation of more efficient electrokinetic phenomena. Faster ICEO and ACEO flows using 3D electrodes [28], and superior filtering efficiencies of DEP using 3D carbon electrodes [21] compared to that generated by planar metal electrodes are some examples. Due to the ready-made pencil-leads being rigid rods, they also served as microchannel molds (a blunt end needle can also be used for this purpose) without any need to use costly fabrication apparatuses such as sputtering machines in clean rooms. Use of these graphite rods as both molds and 3D electrodes enables us to fabricate cylindrical microchannels and apply electric fields at different directions to study the electrokinetic phenomena in polydimethylsiloxane (PDMS)/glass or PDMS/PDMS microfluidic devices. Common conductive rods in microfluidic devices are wires, micro wires [29], and allotropes of carbon such as graphite [30]. Pencil-lead was used here as a highly conductive 3D electrode, since it is accessible and disposable, and can be aligned with an identical pencil-lead mold due to its rigidity. However, this comes with a drawback: pencil-leads are fragile and more likely to break. Here, the molds and electrodes were embedded into an elastomer stamp made of PDMS in a single-step process and the microchannel mold (central lead) was gently removed from the cured PDMS without breakage. The cured PDMS was simply bonded to the glass or PDMS substrate to complete the fabrication process. Electrokinetic phenomena including ICEO, ACEO, and DEP in the pencil-lead-based microfluidic device were illustrated and characterized by micro-particle image velocimetry (micro-PIV) measurement and microscopy imaging. 

## 2. Experimental Methods

### 2.1. Device Fabrication

**Figure 1:**
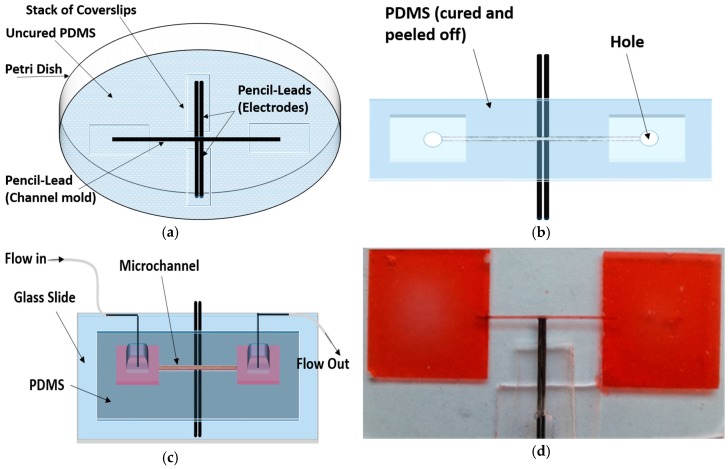
Device fabrication. (**a**) Pencil-leads were positioned on stacks of coverslips sitting on the Petri dish and the polydimethylsiloxane (PDMS ) was cast and cured; (**b**) PDMS including the embedded pencil-leads and coverslips was gently peeled off and the coverslip stacks were removed. The unmolded PDMS was placed on the workbench with the side containing the reservoirs face up, and the central lead (microchannel mold) was removed. The sides of the cured PDMS were cut and removed so that the electrodes could be connected to the power supply. Then, holes were punched into the reservoirs; (**c**) the PDMS containing the microchannel was cleaned, treated and bonded to a treated clean glass slide. Finally, the inlet and outlet tubes were connected to the reservoirs (optionally, the tubes were connected to the reservoirs through small pieces of PDMS bonded to the parts of the device containing the holes in order for inlet and outlet tubes to be fixed tightly in their positions); (**d**) functionality of the microchannel was tested before connecting the tubes.

A simple single-step fabrication method was used here to fabricate a cylindrical channel with 3D bulky electrodes. The microfluidic device consisted of a microchannel, two reservoirs on both ends of the microchannel, and sidewall pencil-lead electrodes. As shown in Figure 1a, four coverslip stacks of the same height (2–5 mm) were placed in a Petri dish. A 0.3 or 0.5 mm pencil-lead (mirochannel mold) was placed at the center of the Petri dish on two coverslip stacks (reservoir molds) from both ends. Sidewall pencil-leads (electrodes) were placed on the other two coverslips. Their positions were adjusted so that they were perpendicular to the microchannel mold. PDMS (Sylgard184 Silicone ElastomerKit, Dow Corning Corp., Freeland, MI, USA) with prepolymer and curing agent weight ratio of 10:1 was cast in the Petri dish covering the molds and electrode array (position of pencil-leads can be readjusted in case they are displaced). PDMS was cured at room temperature after 36 h (curing on a hot plate is quicker, but it may lead to displacement of the leads if they are not fixed). As shown in Figure 1b, the cured PDMS containing the embedded molds and electrodes was gently peeled off, and the coverslip stacks were then removed. The central pencil-lead (mirochannel mold) was peeled off, and the sides of the cured PDMS were cut and removed so that the electrodes could be connected to the power supply. Two holes were then punched on top of the two reservoirs. In case some graphite remained in the microcochannel, it was cleaned and then rinsed with deionized (DI) water. Before bonding, we ensured that the microchannel was not blocked. Figure 1c shows the final fabrication step. The PDMS slab containing the microchannel and a glass slide were cleaned, treated and bonded. Flow inlet and outlet tubes connected to the reservoirs provided the fluid flow through the punched holes (in order for the tubes to be fixed in their positions, they can be connected to the reservoirs through small pieces of PDMS bonded to the reservoirs as shown in Figure 1c). Figure 1d shows the functionality of the microfluidic device before connecting the inlet and outlet tubes. As shown in the figure, food dye was used as a colorant to test the ability of the device in order to properly allow the fluid to pass through the microchannel. One can use multiple pencil-leads to form the mold of a more complicated microfluidic channel, and an optical microscope can be used to align pencil-leads in desired positions. For example, a microchannel with two inlets and outlets is fabricated for the DEP experiments, as shown in Section 3.3. 

### 2.2. Flow Characterization by Micro-PIV

To examine electrokinetic phenomena in the pencil-lead-based microfluidic device, micro-PIV was used to quantify the fluid velocity field within the microchannel. In addition, 1 µm fluorescent micro-beads (Invitrogen, Eugene, OR, USA) suspended in the working fluid were used as flow tracers. Tween 20 (Thermo Fisher Scientific, Fremont, CA, USA) was also added to the suspension for reducing particle adhesion and for stable dispersion of tracer particles [31], and the suspension was sonicated to homogeneously disperse the particles in the fluid prior to the experiment. The microfluidic device was then placed on an inverted optical microscope (TE2000-U, Nikon Eclipse Ti, Nikon Instruments, Tokyo, Japan) equipped with a fluorescence light source and a high-resolution (1376 pixel × 1040 pixel) 15 Hz charge-coupled device (CCD) camera (PowerviewTM, TSI Inc., Shoreview, MN, USA) to capture the flow. Pressure-driven flows of fixed flow rates were provided by an infusion syringe pump (Harvard Apparatus, PHD 2000, Holliston, MA, USA). To generate electrokinetic flows, an AC function generator (Tektronix AFG 3102, Beaverton, OR, USA) was used to apply a sinusoidal AC electric field amplified by a high voltage amplifier (Tegam, Model 2340, Geneva, OH, USA) between the pencil-lead electrodes.

PIVlab [32] (Version 1.41), an open source MATLAB program (R2015b, MathWorks, Natick, MA, USA) for cross-correlation, was used to process the sequence of images taken from the fluorescent tracer particles. First, some image preprocessing such as defining a region of interest (ROI), creating masks and removing background noise prepared the image sequences for velocimetry. PIVlab then used 50% overlapped interrogation windows to quantify the displacement of particles between each pair of images with a certain time interval defined by the camera frame rate, resulting in a velocity vector field for each pair. PIVlab was able to post-process the obtained vector fields including interpolation of missing data, outlier removal and data smoothing as necessary. Ensemble-averaging the velocity vectors of all image pairs at each point resulted in the final averaged velocity vector field. 

**Figure 2:**
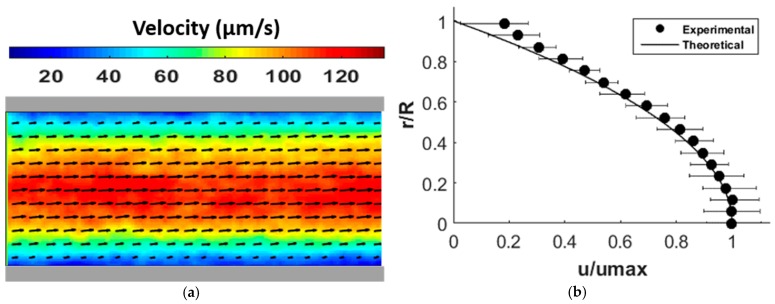
(**a**) Velocity distribution of pressure-driven flow with a volume flow rate of 12 μL/h through a microchannel 0.3 mm in diameter; (**b**) validation of the pressure-driven velocity field in (**a**).

The micro-PIV measurement was validated by a steady pressure-driven flow passing through the cylindrical microchannel, which was provided by the syringe pump at a fixed flow rate. DI water was used as the working fluid and 1 µm carboxylate-modified fluorescent micro-beads suspended in the DI water were used as flow tracers. Figure 2a shows the velocity vector field of pressure-driven flow at a volume rate of 12 μL/h. Figure 2b demonstrates the validity of the velocity distribution when compared to the parabolic velocity profile of an incompressible laminar pipe flow with no slip on walls. *R* and *u*_max_ are the microchannel radius and the averaged maximum velocity (in the neighborhood of the centerline). The experimental data points show the average dimensionless velocities at each radial position. The error bars show the minimum and maximum dimensionless velocities of each radial position at different cross sections along the flow direction. As expected, micro-PIV provided less accurate information about velocities in the vicinity of the microchannel wall. Interrogation windows overlapped with the stationary surface regions (wall) is a source of this error [33].

## 3. Results

**Figure 3:**
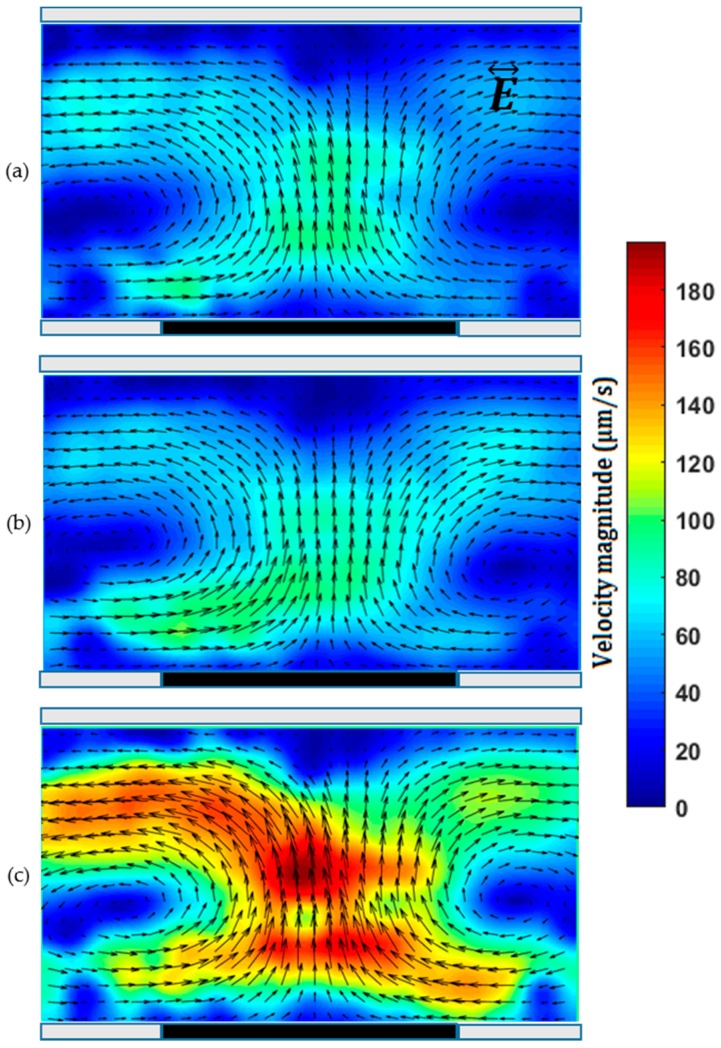
Induced-charge electroosmosis (ICEO) flow in front of a 0.5 mm pencil-lead in a channel of 0.5 mm diameter under an applied alternating current (AC) voltage of (**a**) 400 V_p-p_; (**b**) 450 V_p-p_; (**c**) 500 V_p-p_ with a frequency of 500 Hz between two pencil-lead electrodes positioned about 11 mm apart in reservoirs.

**Figure 4:**
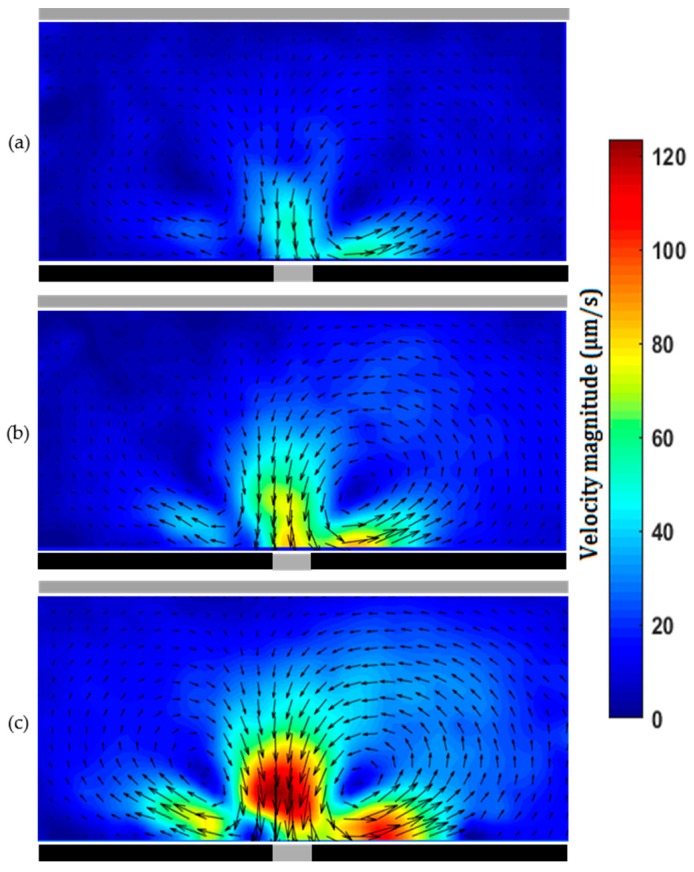
Alternating current electroosmosis (ACEO ) flow of 1 mM KCl solution between two parallel 0.3 mm pencil-lead electrodes with a gap of 40 µm in a microchannel of 0.3 mm diameter. AC voltage of (**a**) 30 V_p-p_; (**b**) 35 V_p-p_; (**c**) 40 V_p-p_ with a frequency of 50 kHz.

**Figure 5:**
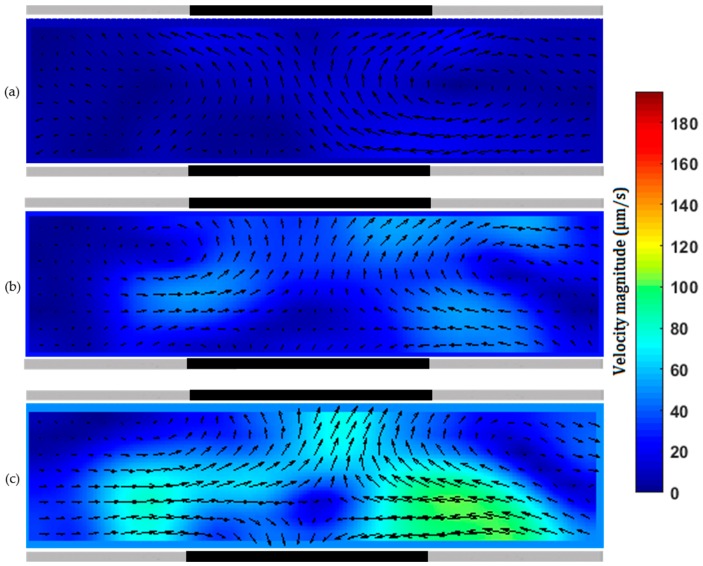
ACEO flow of 1 mM KCl solution between two 0.5 mm pencil-lead electrodes facing each other in a microchannel of 0.3 mm diameter. AC voltage of (**a**) 30 V_p-p_; (**b**) 35 V_p-p_; (**c**) 40 V_p-p_ with a frequency of 50 kHz.

**Figure 6:**
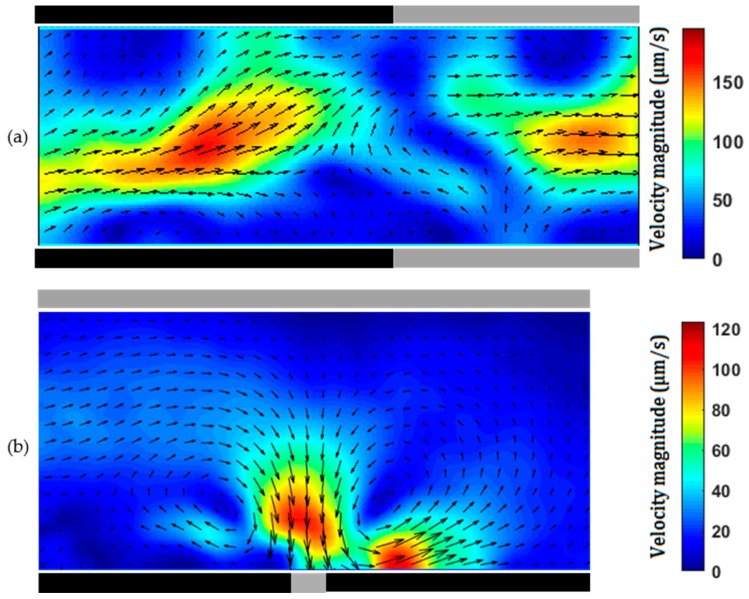
(**a**) Combination of a pressure-driven flow with a volume flow rate of 12 μL/h and ACEO flow between two 0.5 mm pencil-lead electrodes facing each other in a microchannel of 0.3 mm diameter with an AC voltage of 40 V_p-p_ at 50 kHz; (**b**) combination of a pressure-driven flow with a volume flow rate of 6 μL/h and ACEO flow in a microchannel of 0.3 mm diameter with two parallel 0.3 mm pencil-lead electrodes between which the gap is 40 µm. The applied AC voltage on the electrodes is 40 V_p-p_ at 50 kHz.

The main objective of the present study was simple fabrication of microchannels with embedded 3D sidewall electrodes (shown in black in Figure 3, Figure 4, Figure 5 and Figure 6) to generate electrokinetic transport phenomena. We demonstrated ICEO, ACEO and DEP with the devices fabricated with the developed method. Since the pencil-lead is relatively big (0.3 to 0.5 mm), multiple experiments can be conducted in the same device. 

### 3.1. ICEO

To achieve ICEO, the sidewall electrodes made of pencil-leads are floating, and an external AC electric field is applied through two pencil-leads inserted into the two holes of the reservoirs. In most of the existing studies, ICEO was typically induced in the vicinity of a thin layer of floating polarizable surface, such as a gold film, fabricated by an electrical sputtering system [34]. In addition to the high cost, the thin metal layer also suffers from a short life time due to its small thickness [35]. Here, highly conductive and durable bulk pencil-leads are used as 3D polarizable objects to overcome these deficiencies. 

Here, 1 µm carboxylate-modified fluorescent micro-beads were used to demonstrate ICEO flow of 1 mM KCl solution in a fabricated microchannel with a side wall pencil-lead. A pencil-lead of 0.5 mm in diameter was used as the microchannel mold, and only one pencil-lead of 0.5 mm in diameter was embedded as the side wall electrode. Figure 3 shows the base of the pencil-lead (as a conductive surface) as part of the sidewall of a microchannel where ICEO flow was generated by applying an AC field between the two reservoirs. Video S1 (in Supplementary Information) shows the ICEO results under different applied voltages. For a floating electrode, a symmetric ICEO flow is expected [36]. Briefly, when the electric field is applied, part of the floating lead is positively charged and the rest is negatively charged, and an induced electrical double layer is formed in the vicinity of the floating pencil-lead. The interaction between the applied electric field and the net charges within the induced electrical double layer creates an electrostatic force and accordingly an ICEO flow. Due to the presence of opposite charges, two eddies with opposite flow directions are formed near the floating lead, as shown in Figure 3. Figure 3a–c also shows that the ICEO flow speed is highly dependent on electric field strength, and the ICEO flow is nearly symmetric with respect to the center of the lead. One can have multiple sidewall pencil-leads to further enhance the ICEO flow within the microchannel. By using pencil-leads of a few hundred microns, one can thus obtain results related to ICEO flow around one or multiple cylinders shown by Canpolat et al. [31,37], who fabricated the cylindrical rod by the electroplating technique. In contrast, use of the commercial pencil-lead is simpler and cheaper.

### 3.2. ACEO

Different from the above ICEO flow where the sidewall electrode(s) are just floating, a potential applied to the sidewall electrode is used for generating ACEO flow. To achieve ACEO flow, we fabricate a microchannel with a pair of sidewall pencil-lead electrodes positioned either side-by-side (Figure 4) or face-to-face (Figure 5). Video S2 (in Supplementary Information) shows the ACEO flow around a pair of side-by-side pencil-leads under different voltages. The pair of pencil-leads connect to the AC function generator amplified by the high-voltage amplifier. Different AC voltages of fixed frequency were applied to the pair of sidewall pencil-lead electrodes. ACEO flow results from the interaction between the applied electric field and the net charges within the electrical double layer of each working electrode. The major advantage of using simple 3D electrodes as bulk polarizable objects over planar conductive surfaces is the enhancement in ACEO flow velocity [38]. The introduced fabrication method can also be used to provide stepped 3D pencil-lead electrodes. The flow rate and frequency range capable of ACEO pumping were enhanced significantly when planar electrodes were replaced by stepped 3D electrodes [39]. Figure 4 and Figure 5 show ACEO flow of a 1 mM KCl solution (ACEO is limited to electrolytes with low ionic strength [40]) under different AC fields applied across a pair of side-by-side and face-to-face pencil-lead electrodes, respectively. It should be noted that, for the 1 mM KCl solution with molar electric conductivity of 146.88 × 10^-4^ Sm^2^/mol [41] (conductivity of 0.014 S/m), ACEO dominated other electrohydrodynamic effects such as dielectrophoresis and electrothermal effects in the microchannel due to the low frequency employed here [42].

In case of parallel electrodes shown in Figure 4, the ICEO flow near one electrode is stronger than that near the other, and the flow is not symmetric with respect to the center of the gap between the two electrodes. This is the minimum gap achieved through the fabrication process and was measured by the microscope. This gap can be adjusted under the microscope at the beginning of the fabrication process. For parallel planar electrodes, a symmetric ACEO flow is expected [43]; however, the cross sections of both the microchannel and each electrode are circular in our device, which is the main reason for the asymmetric ICEO flow observed here. Comparison of velocity fields around the vortices shown in Figure 4a–c depicts that the strength of the ACEO flow increases approximately quadratically with increase in the applied AC voltage, which qualitatively agrees with the results from the literature [44]. In the experiments, we found that drastic decrease in frequency (from the range of kHz to Hz) or increase in the AC voltage (>60 V_p-p_) increased the risk of electrolysis, resulting in the creation of bubbles around the electrodes. 

For the case of face-to-face pencil-leads (Figure 5), at a relatively low AC voltage, the flow is mainly directed from one electrode towards the other and two opposite circulations are formed, as shown by Figure 5a. As the voltage increases, the flow is directed toward each electrode, which is clearly shown in Figure 5c. As the voltage increases, both the magnitude and the region of the ACEO flow increase. The ACEO flow velocity with face-to-face electrodes is stronger than that with side-by-side electrodes. In both cases, increasing the frequency (to 1 MHz) in the reported range of applied voltages did not show a notable change in flow velocity. However, the ACEO flow decayed drastically when the frequency exceeded 2 MHz. 

One can use a syringe pump to pump sample through the microchannel with pencil-lead sidewall electrodes. Since the Reynolds numbers in microchannels are very small, the flow is laminar and mixing is challenging. One can apply AC voltage through a pair of pencil-lead sidewall electrodes to generate secondary ACEO flow, which will perturb the main pressure-driven flow. Figure 6 shows the superposition of the pressure-driven and ACEO flows using both electrode configurations. Without the ACEO flow, the velocity profile in the pressure-driven flow is nearly parabolic, as shown in Figure 2. Clearly the pressure-driven flow is perturbed in the region with the ACEO flow, and the formed vortex can be used for mixing enhancement. To create chaotic advection for mixing enhancement, one can have multiple pairs of sidewall electrodes made of pencil-lead, and alternately apply an AC voltage to the sidewall electrodes [45,46]. In addition to the combination of the pressure-driven and ACEO flows for mixing enhancement, one can also apply a DC electric field through two pencil-leads inserted into the two reservoirs to generate electroosmotic flow. Under DC field, ICEO flow also forms on the floating sidewall pencil-leads. The formed secondary ICEO flow can perturb the main electroosmotic flow for mixing enhancement. 

### 3.3. DEP

**Figure 7:**
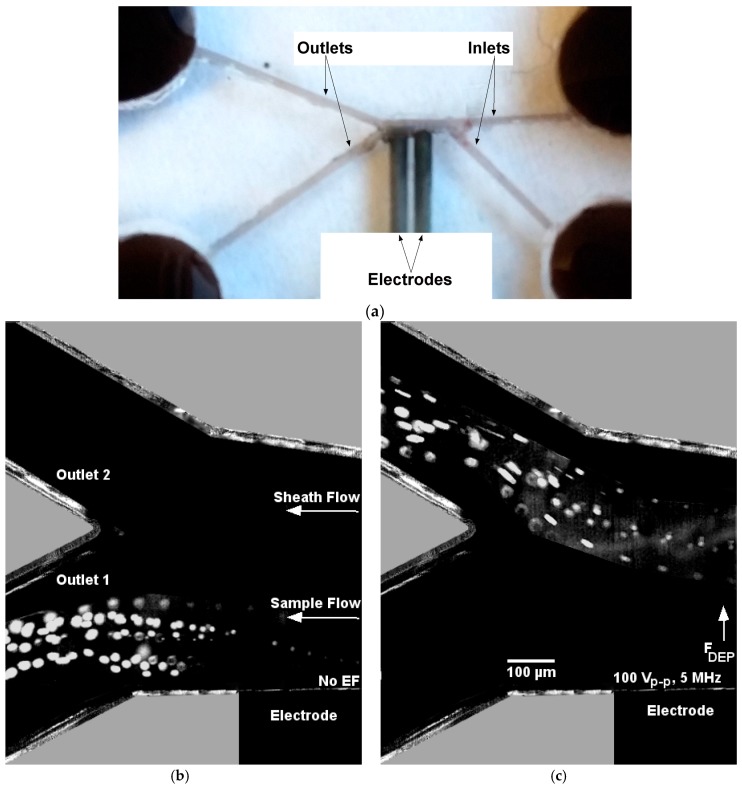
Microfluidic device for dielectrophoresis (DEP)-based particle switching. (**a**) A fabricated device having two inlets and outlets. Parallel pencil-lead electrodes were aligned near one outlet to apply the desired AC field in the microchannel; (**b**) superimposed trajectories of 10 µm fluorescent particles suspended in deionized (DI) water exiting the microchannel under no electric field; (**c**) DEP force generated by AC voltage of 100 V_p-p_ at a frequency of 5 MHz repelled the particles to outlet 2.

DEP refers to the migration of polarizable objects in an aqueous solution under a spatially non-uniform electric field [7]. Complex Clausius–Mossotti (CM) factor (a function of the ratio of the particle and electrolyte polarizability [47]) determines whether the particles experience positive or negative DEP. Use of the pencil-lead sidewall electrodes enables us to locally apply a voltage and generate spatially non-uniform AC field, and accordingly a DEP force acting on the particle. We used 10 µm polystyrene fluospheres to demonstrate DEP here. We used the method described in Section 2.1 to fabricate a microfluidic device similar to that fabricated by Puttaswamy et al. [48]. The main microchannel has two inlets on one side and two outlets at the other end, as shown in Figure 7a. Two parallel pencil-leads of 0.5 mm diameter are placed near outlet 1, and the gap between the two pencil-lead electrodes is about 200 µm as positioned under microscope. Figure 7a shows the functionality of the device. Sheath flow with a flow rate of 4.5 µL/min pumps pure DI water without particles through one inlet, and the other inlet pumps DI water mixed with 10 µm polystyrene fluospheres at a flow rate of 1.6 µL/min. Without applying a voltage to the pencil-lead electrodes, particles flow through outlet 1, and no particles exit from outlet 2, as shown in Figure 7b. When an AC voltage of 100 V_p-p_ with a frequency of 5 MHz is applied between the two pencil-leads, particles flow to the second outlet, as shown in Figure 7c. Video S3 (in Supplementary Information) shows the particles’ motion before and after application of the electric field. Under our experimental condition, the CM factor is negative, confirming that the particle experiences negative DEP. In addition, due to the high frequency, ACEO is negligible. Due to negative DEP, particles are pushed away from the electrodes, and then are carried by the faster sheath flow to outlet 2. Since the DEP force is proportional to the particle size, the deflection distance from the wall depends on the particle size. Thus, one can add more outlets to the device to achieve DEP-based particle separation by size. 

## 4. Discussion

The demonstrated electrokinetic phenomena using our cheap and straightforward device fabrication method show a promising way for manipulating fluids and particles beneficial for various microfluidic applications. For instance, 3D pencil-leads embedded into PDMS-based microfluidic devices could offer highly polarizable floating electrodes for generating strong ICEO flow, yet ACEO flow created under low voltages between these low cost graphite rods could efficiently mix fluids. DEP control of particles under non-uniform AC fields generated by these aligned highly conductive pencil-leads is able to achieve particle focusing, separation and trapping. Obviously, the pencil-lead-based microfluidic devices also have certain disadvantages. Since the pencil-lead is rigid, it can only fabricate straight microchannels of a few hundreds microns in diameter. The size and shape of the microchannel and electrode are constrained by that of the pencil-lead. The position and alignment of pencil-leads are not as accurate as in conventional photolithography techniques. However, they provide a cheap and quick method to fabricate a microfluidic device with 3D electrodes to generate various electrokinetic phenomena for various applications without the use of expensive fabrication facilities. 

## 5. Conclusions

A pencil-lead-based microfluidic channel was developed and tested by implementation of multiple electrokinetic phenomena. The microchannel mold was a pencil-lead instead of the typical mold made of SU-8 photoresist, and pencil-leads also constituted the 3D sidewall electrodes. The use of these accessible molds and electrodes eliminates time-consuming and expensive conventional fabrication procedures such as coating, UV exposing, developing and sputtering. This facile, low cost technique allows one to fabricate microchannels of different dimensions using different diameter pencil-leads as well as configure different geometries by using multiple pencil-leads as sidewall electrodes at various positions. As shown in this paper, one can easily implement various electrokinetic phenomena including EOF, ICEO, ACEO and DEP in the fabricated device. One can also combine pressure-driven flow with the aforementioned electrokinetic phenomena to manipulate samples for various microfluidic applications. A significant advantage of using these bulk electrodes is to increase the electrokinetic flow velocity. Micro-PIV measurements of the phenomena illustrated in this paper underscore the capability of the fabricated devices for several microfluidic applications such as local mixing, fluid pumping and particle manipulation.

## Supplementary Materials

The following are available online at www.mdpi.com/2072-666X/7/12/235/s1, Video S1: ICEO flow described in Section 3.1, Video S2: ACEO flow described in Section 3.2, Video S3: DEP described in Section 3.3.
