# Supplementary figures and images for: Electrokinetic Phenomena in Pencil Lead-Based Microfluidics
Source: Micromachines (Basel). 2016 Dec 15;7(12):235. doi: 10.3390/mi7120235 (PMC6190385; doi:10.3390/mi7120235)

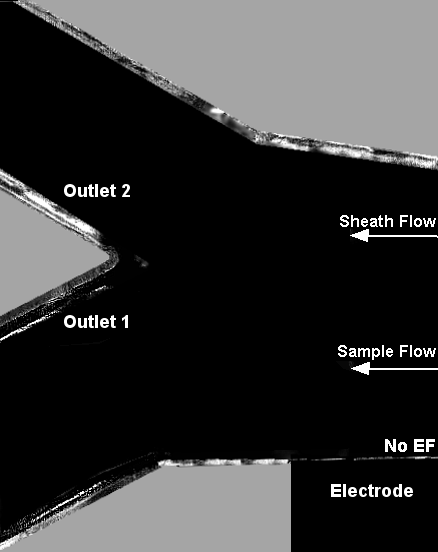

Supplement: Supplementary file 1 [file micromachines-07-00235-s001.zip › VideoS3.gif]
